# Supplementary material for: HDAC Inhibitory and Anti-Cancer Activities of Curcumin and Curcumin Derivative CU17 against Human Lung Cancer A549 Cells
Source: Molecules. 2022 Jun 22;27(13):4014. doi: 10.3390/molecules27134014 (PMC9268269; doi:10.3390/molecules27134014)
Supplement: Supplementary file 1 [file molecules-27-04014-s001.zip › molecules-1739774-supplementary.pdf]

Supplementary materials

Article

HDAC inhibitory and anti-cancer activities of curcumin and curcumin derivative CU17 against human lung cancer A549 cells

Narissara Namwan[1], Gulsiri Senawong[1], Chanokbhorn Phaosiri[2], Pakit Kumboonma[3], La-or Somsakeesit[4], Chadaporn Leerat[1] and Thanaset Senawong[1,*]

Table S1. Interaction of CU in the HDAC active sites.

HDACs	Binding interaction (H-bond)
HDAC1	His178 (2.11 Å), Tyr204 (2.84 Å), Ser267 (2.56 Å, 2.77 Å), Asn275 (2.83 Å) , Ser348 (2.10 Å)
HDAC2	Zn (2.30 Å), Asp104 (2.81 Å), Cys156 (2.85 Å), His183 (2.34 Å), Gln265 (2.97 Å), Gly305 (2.06 Å)
HDAC3	Zn (2.70 Å), Asp93 (2.88 Å), Gly132 (2.66 Å), Gly143 (2.95 Å, 3.33 Å), Leu266 (2.84 Å, 3.00 Å), Gly296 (2.79 Å)
HDAC4	Zn (2.20 Å), Lys20 (2.92 Å), Arg37 (1.77 Å), Asn119 (1.98 Å), Glu120 (1.58 Å), His158 (2.81 Å), Tyr170 (2.08 Å), Asp196 (2.70 Å)
HDAC6	Zn (4.0 Å), Trp261 (3.51 Å), His232 (3.04 Å), Pro329 (2.81 Å)), Tyr363 (3.81 Å)
HDAC7	Arg731 (1.97 Å), Leu810 (3.81 Å), His709 (3.98 Å), His843 (3.46 Å)
HDAC8	Zn (3.1 Å), Gly151 (2.29 Å), His143 (2.69 Å), His180 (4.31 Å) , Pro205 (2.04 Å), Tyr306 (2.04 Å)

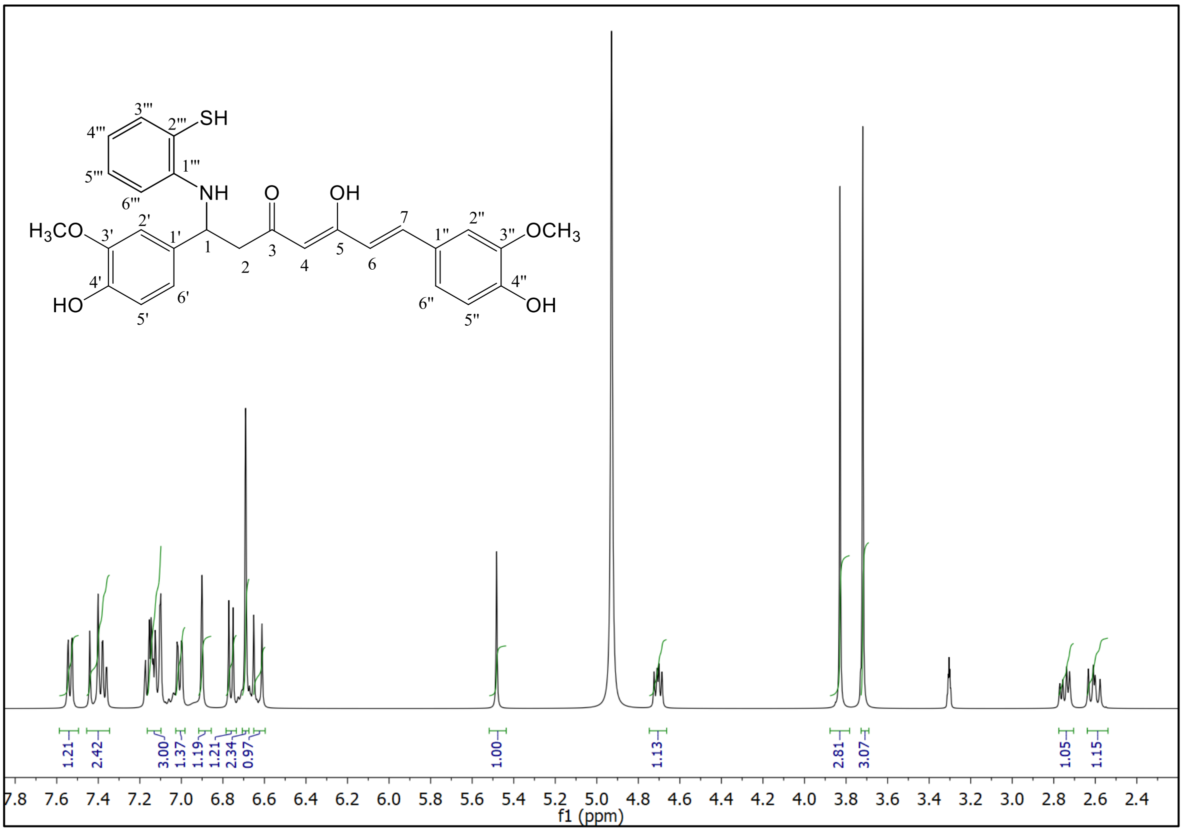

Figure S1. ^{1}H NMR spectrum of CU17.

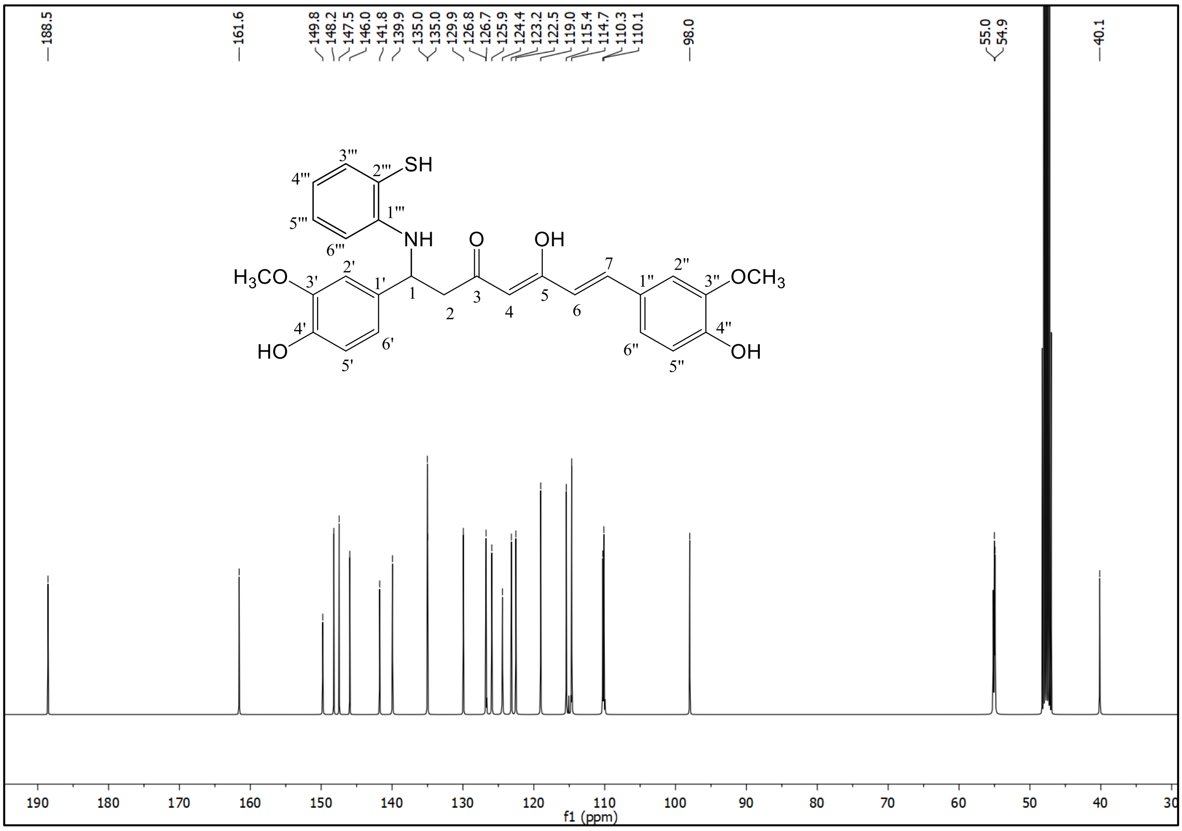

Figure S2. ^{13}C NMR spectrum of CU17.

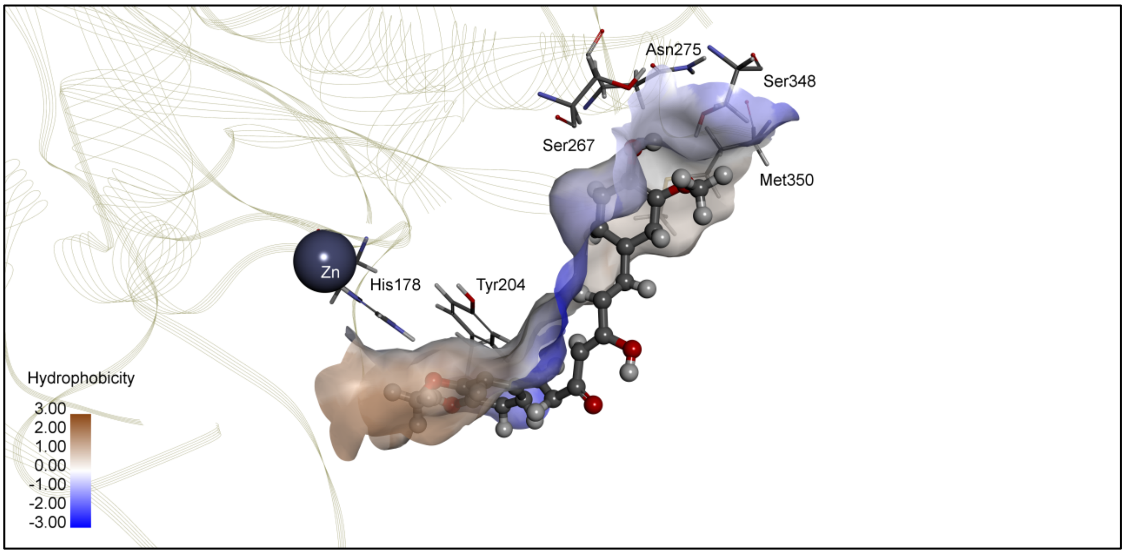

Figure S3. The interaction between CU and the active site of HDAC1.

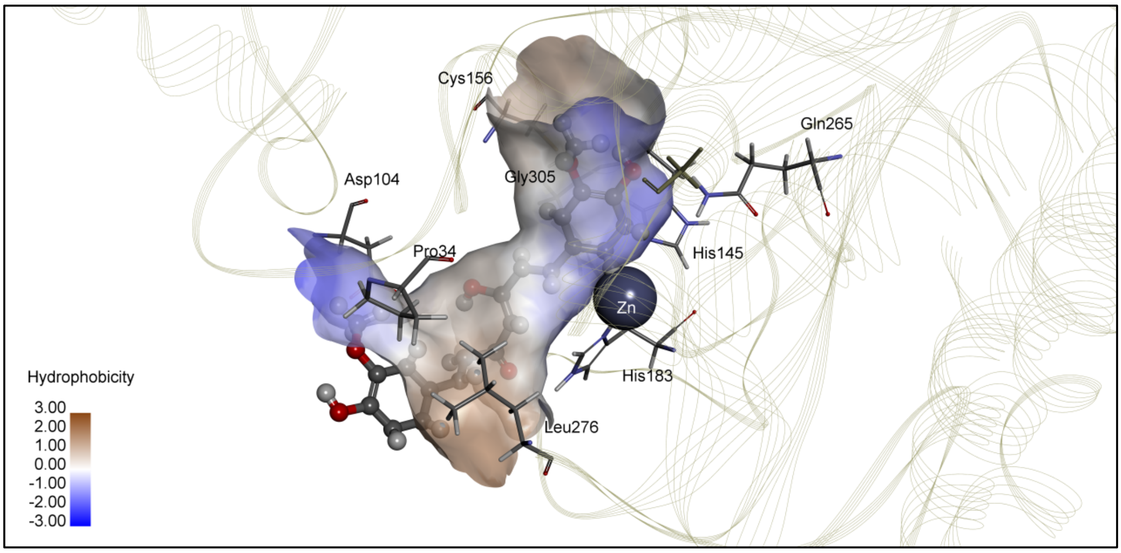

Figure S4. The interaction between CU and the active site of HDAC2.

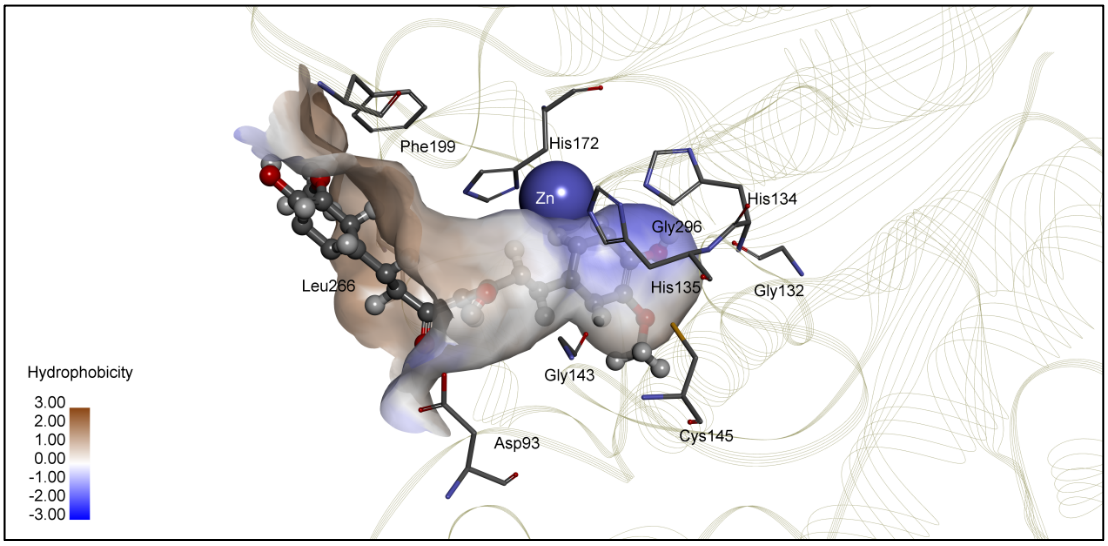

Figure S5. The interaction between CU and the active site of HDAC3.

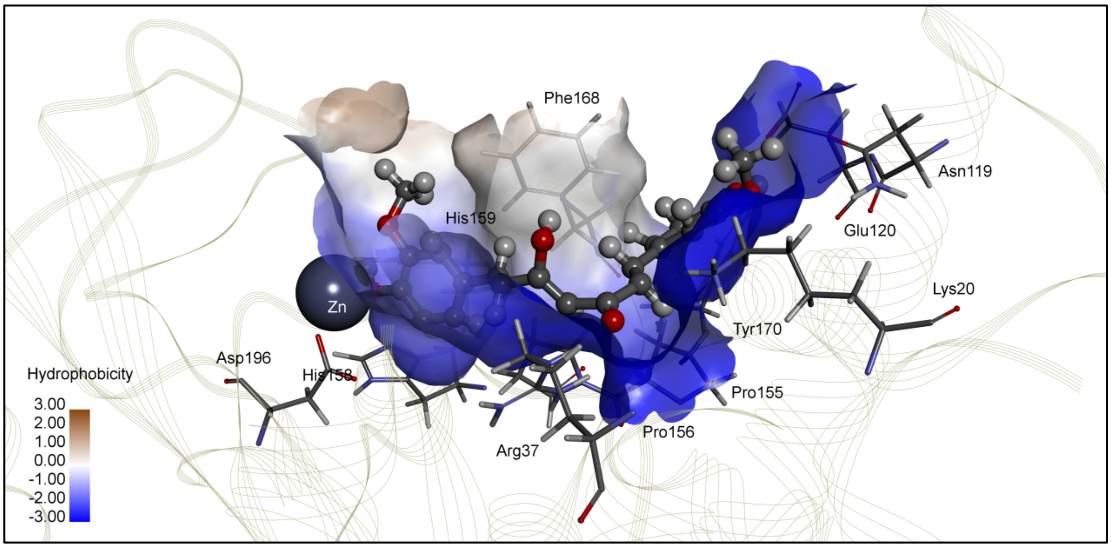

Figure S6. The interaction between CU and the active site of HDAC4.

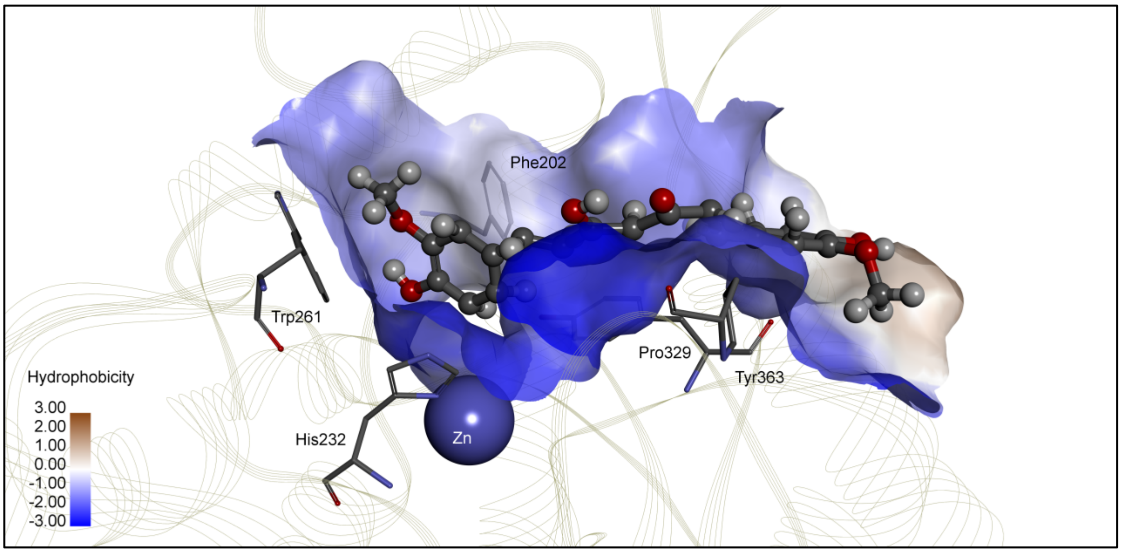

Figure S7. The interaction between CU and the active site of HDAC6.

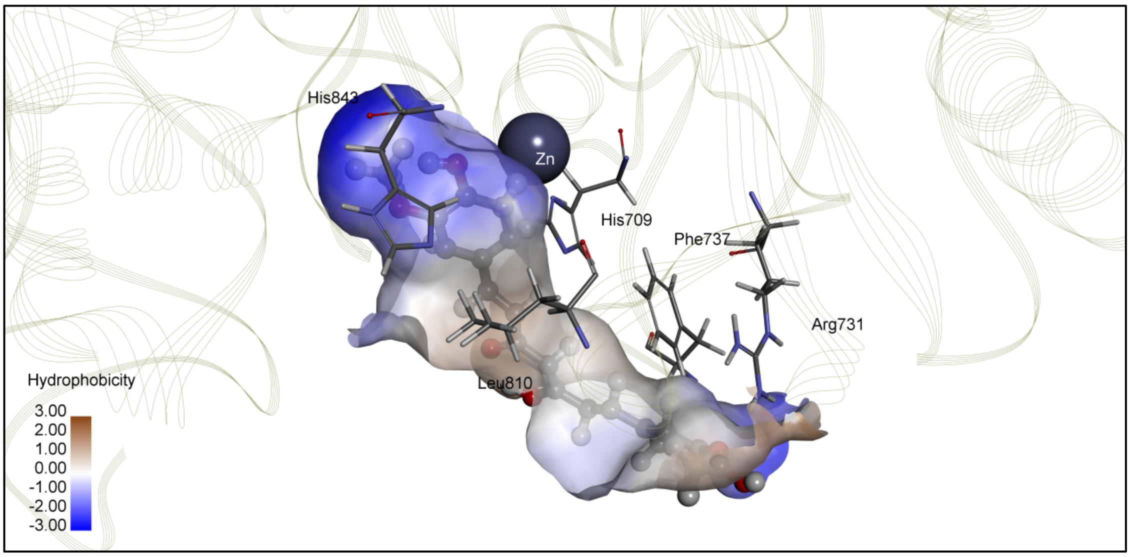

Figure S8. The interaction between CU and the active site of HDAC7.

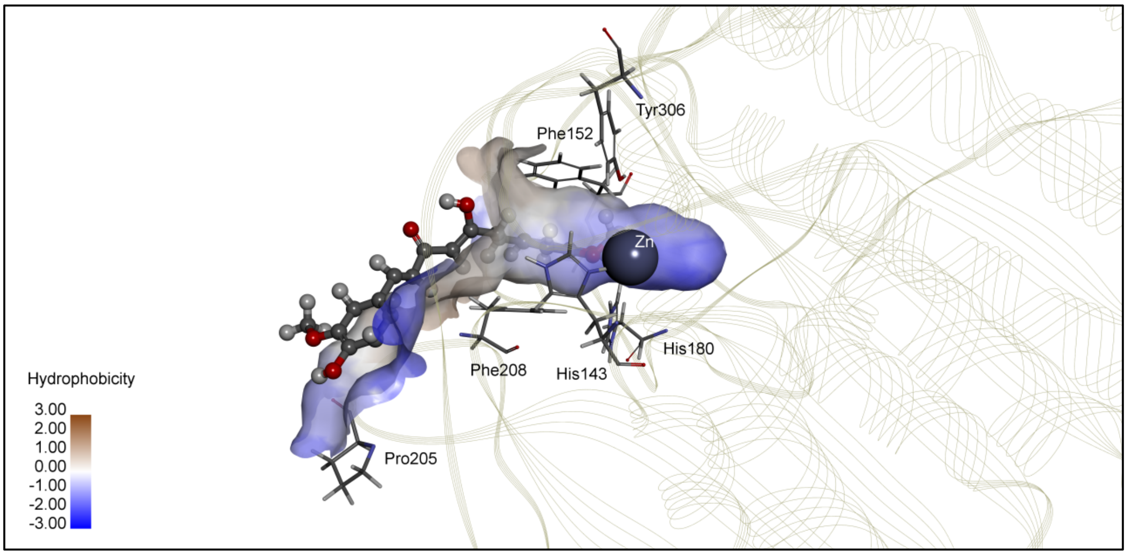

Figure S9. The interaction between CU and the active site of HDAC8.

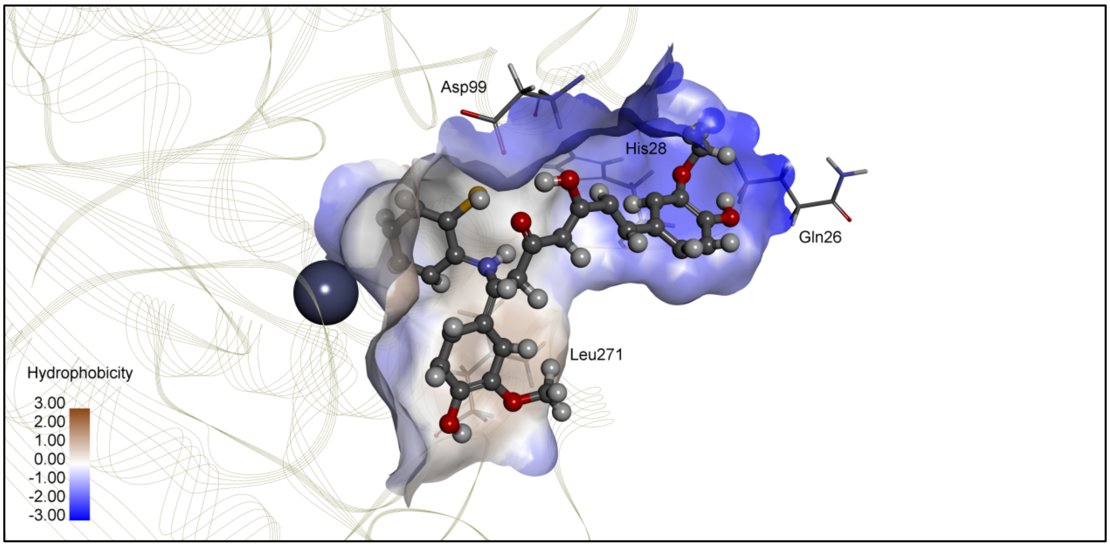

Figure S10. The interaction between CU17 and the active site of HDAC1.

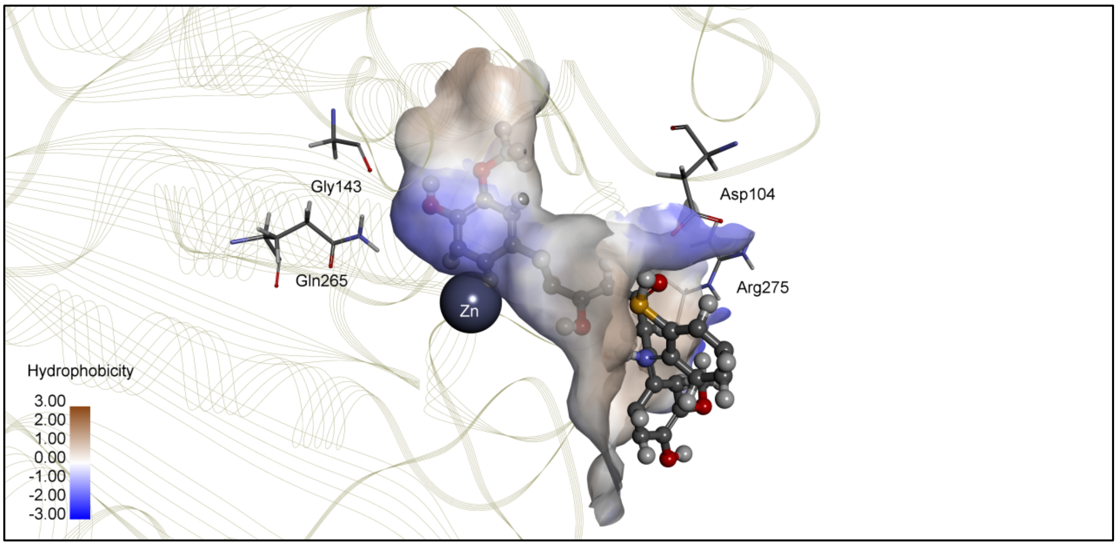

Figure S11. The interaction between CU17 and the active site of HDAC2.

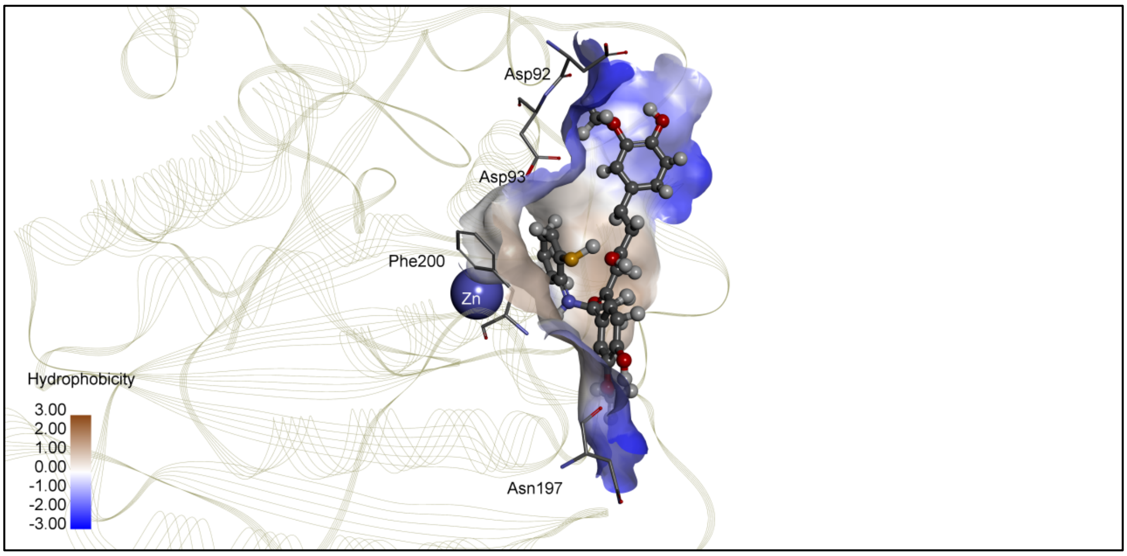

Figure S12. The interaction between CU17 and the active site of HDAC3.

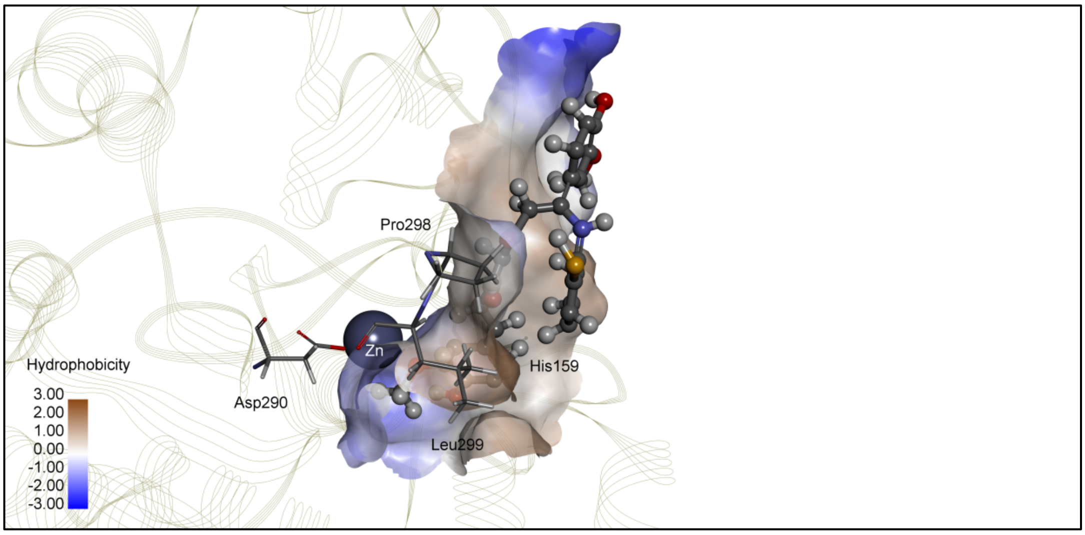

Figure S13. The interaction between CU17 and the active site of HDAC4.

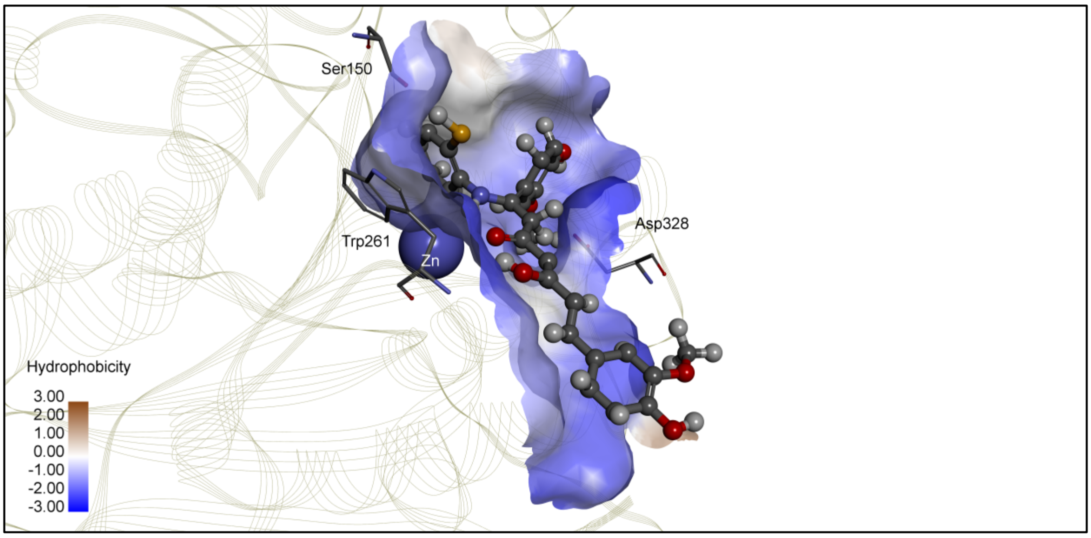

Figure S14. The interaction between CU17 and the active site of HDAC6.

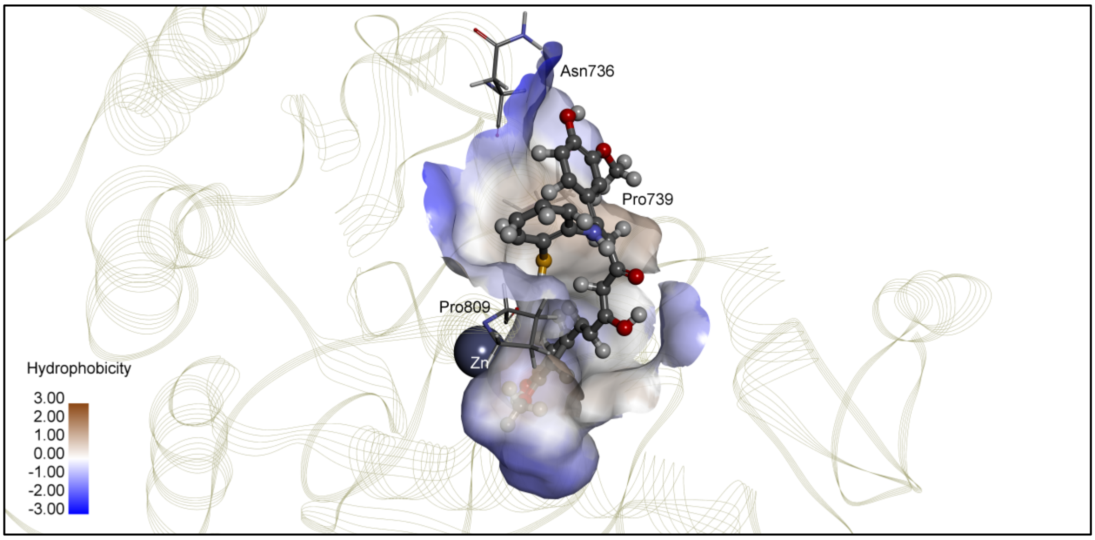

Figure S15. The interaction between CU17 and the active site of HDAC7.

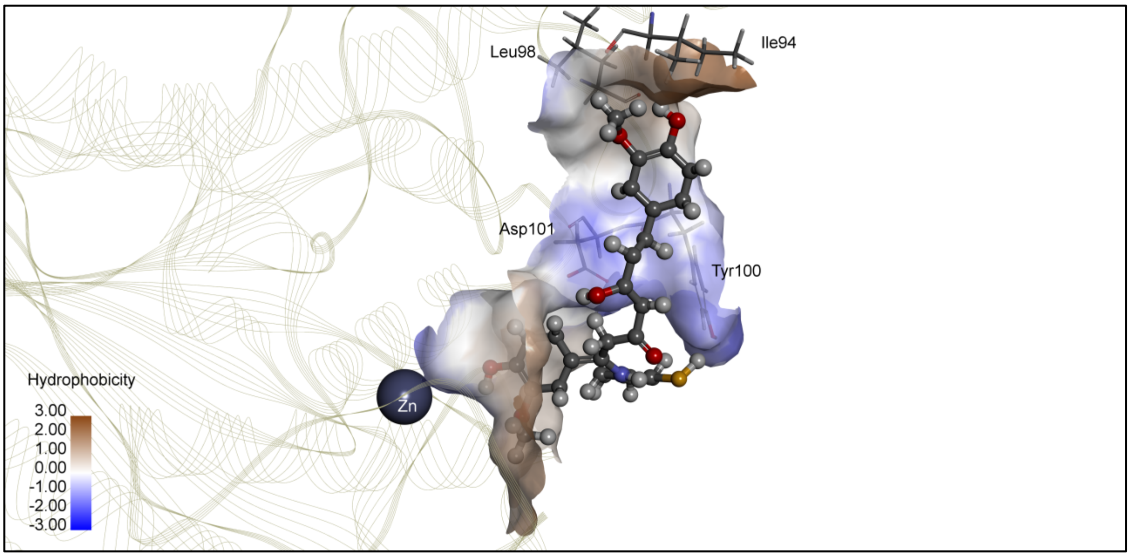

Figure S16. The interaction between CU17 and the active site of HDAC8.
